# Strain, Soil-Type, Irrigation Regimen, and Poultry Litter Influence *Salmonella* Survival and Die-off in Agricultural Soils

**DOI:** 10.3389/fmicb.2021.590303

**Published:** 2021-03-16

**Authors:** Cameron A. Bardsley, Daniel L. Weller, David T. Ingram, Yuhuan Chen, David Oryang, Steven L. Rideout, Laura K. Strawn

**Affiliations:** ^1^Department of Food Science and Technology, Eastern Shore Agricultural Research and Extension Center, Virginia Tech, Painter, VA, United States; ^2^Department of Environmental and Forest Biology, SUNY College of Environmental Science and Forestry, Syracuse, NY, United States; ^3^Center for Food Safety and Applied Nutrition, US Food and Drug Administration, College Park, MD, United States; ^4^School of Plant and Environmental Sciences, Eastern Shore Agricultural Research and Extension Center, Virginia Tech, Painter, VA, United States

**Keywords:** *Salmonella*, irrigation, time to harvest interval, biological soil amendments of animal origin, strain variability, survival, poultry litter, die-off rate

## Abstract

The use of untreated biological soil amendments of animal origin (BSAAO) have been identified as one potential mechanism for the dissemination and persistence of *Salmonella* in the produce growing environment. Data on factors influencing *Salmonella* concentration in amended soils are therefore needed. The objectives here were to (i) compare die-off between 12 *Salmonella* strains following inoculation in amended soil and (ii) characterize any significant effects associated with soil-type, irrigation regimen, and amendment on *Salmonella* survival and die-off. Three greenhouse trials were performed using a randomized complete block design. Each strain (~4 log CFU/g) was homogenized with amended or non-amended sandy-loam or clay-loam soil. *Salmonella* levels were enumerated in 25 g samples 0, 0.167 (4 h), 1, 2, 4, 7, 10, 14, 21, 28, 56, 84, 112, 168, 210, 252, and 336 days post-inoculation (dpi), or until two consecutive samples were enrichment negative. Regression analysis was performed between strain, soil-type, irrigation, and (i) time to last detect (survival) and (ii) concentration at each time-point (die-off rate). Similar effects of strain, irrigation, soil-type, and amendment were identified using the survival and die-off models. Strain explained up to 18% of the variance in survival, and up to 19% of variance in die-off rate. On average *Salmonella* survived for 129 days in amended soils, however, *Salmonella* survived, on average, 30 days longer in clay-loam soils than sandy-loam soils [95% Confidence interval (CI) = 45, 15], with survival time ranging from 84 to 210 days for the individual strains during daily irrigation. When strain-specific associations were investigated using regression trees, *S.* Javiana and *S.* Saintpaul were found to survive longer in sandy-loam soil, whereas most of the other strains survived longer in clay-loam soil. *Salmonella* also survived, on average, 128 days longer when irrigated weekly, compared to daily (CI = 101, 154), and 89 days longer in amended soils, than non-amended soils (CI = 61, 116). Overall, this study provides insight into *Salmonella* survival following contamination of field soils by BSAAO. Specifically, *Salmonella* survival may be strain-specific as affected by both soil characteristics and management practices. These data can assist in risk assessment and strain selection for use in challenge and validation studies.

## Introduction

Past reports estimate that *Salmonella* is the leading cause of bacterial foodborne disease outbreaks linked to fresh produce in the United States (US) and Europe (Hanning et al., 2009; Callejón et al., 2015). Fruits and vegetables are particularly problematic as an etiological vehicle due to the lack of a kill step between harvest and consumption that would eliminate or reduce pathogen loads. Thus, there is particular interest in preventing *Salmonella* contamination of fresh produce, including in the pre-harvest environment. As illustrated by past *Salmonella* outbreaks in Delaware, and the eastern shore of Maryland and Virginia (i.e., the Delmarva; Greene et al., 2008; Hanning et al., 2009; Painter et al., 2013; Bell et al., 2015; CDC, 2016; Gu et al., 2018; Truitt et al., 2018), there are multiple mechanisms whereby produce can be contaminated in the farm environment. In response to the aforementioned outbreaks, multiple studies investigated potential sources of the outbreak serovar in the Delmarva, and identified wildlife (Gruszynski et al., 2014), irrigation water (Greene et al., 2008; Hanning et al., 2009), and the use of biological soil amendments of animal origin (BSAAO) as potential *Salmonella* sources (CDC, 2016; Gu et al., 2019). Moreover, studies in other produce-growing regions (e.g., California, New York, and Florida) have also detected *Salmonella* in agricultural field soils (Gorski et al., 2011; Strawn et al., 2013a,b, 2014; Bell et al., 2015), irrigation water (McEgan et al., 2013; Cooley et al., 2014; Weller et al., 2015, 2020), and BSAAOs (Gu et al., 2019). As such, preventing pre-harvest produce contamination is a priority for industry and public health professionals.

Since the pre-harvest environment represents a key route for *Salmonella* contamination of fresh produce, understanding pathogen ecology in pre-harvest environments, including factors affecting pathogen survival and growth, is key to developing effective food safety risk mitigation strategies. For example, past studies (Chandler and Craven, 1980; Danyluk et al., 2007; Underthun et al., 2018; Jechalke et al., 2019) have suggested that *Salmonella* can survive for long periods in soils and survival was dependent on soil characteristics (e.g., moisture, composition, and temperature). Specifically, the use of BSAAOs appears to facilitate *Salmonella* survival (Baloda et al., 2001; Holley et al., 2006; You et al., 2006; Nyberg et al., 2010; Ongeng et al., 2011; Hruby et al., 2018; Shah et al., 2019). For example, a study conducted in Iowa (Hruby et al., 2018) detected *Salmonella* in soil samples collected from plots amended with poultry litter approximately 1 year after BSAAO application. Similarly, a study (You et al., 2006) recovered *Salmonella* from bovine manure-amended soils 332 days after application in the laboratory (controlled temperature and moisture). These findings are concerning from a food safety stand-point because *Salmonella* in soils contaminated by BSAAO can transfer to the harvestable portion of crops (Natvig et al., 2002; Islam et al., 2004a,b; Franz et al., 2005; Kenney et al., 2006; Oni et al., 2015; Kumar et al., 2017). Cessation of BSAAO application to meet food safety concerns is impractical since BSAAOs, such as poultry litter, are economical, organic fertilizers whose application promotes soil health and fertility (Watts et al., 2010; Gu et al., 2019). As such, additional research is needed to better understand *Salmonella* dynamics following BSAAO amendment, and provide growers with specific guidance on how to best manage food safety hazards in BSAAOs.

Recognizing the food safety risks associated with as well as the economic importance of BSAAOs, stakeholders have developed recommendations on intervals between application of untreated BSAAO and harvest. For example, the United States Department of Agriculture (USDA) National Organic Program recommends intervals of at least 120 days between manure application and harvest if the edible/harvestable portion of the produce has direct contact with the soil (USDA, 2015, 2020). However, it is unclear (i) if existing data support the National Organic Program guidelines and (ii) if a one-size-fits all guideline is appropriate for all commodities, management practices (e.g., type of BSAAO used and irrigation regimen), produce-growing regions, and even fields within a single operation. Indeed, past studies have shown that soil characteristics, environmental and laboratory conditions (e.g., temperature), and amendment type can all affect *Salmonella* survival in soil (Himathongkham et al., 1999; You et al., 2006; Semenov et al., 2009; Ramos et al., 2019). Similarly, past studies (Strawn et al., 2014; Weller et al., 2015; Sharma et al., 2020) indicates that factors that vary between produce-growing regions, between farms within the same region, and between fields on the same farm can affect pathogen dynamics in farm environments.

Furthermore, a large variety of *Salmonella* strains has been identified in different types of manure, including poultry manure (Elgroud et al., 2009; Alali et al., 2010; Chinivasagam et al., 2010; Donado-Godoy et al., 2012; Jay-Russell et al., 2018a,b). For example, one study (Alali et al., 2010) identified 70 different strains of *Salmonella* in seven broiler poultry farms. Another study (Jay-Russell et al., 2018b) reported that, among 66 *Salmonella* isolates from untreated poultry manure samples obtained in the western US over a year, whole genome sequencing data showed 12 *Salmonella* serotypes or serotype clusters. While several studies (Franz et al., 2008, 2011) performed in laboratory settings, using cattle manure-amended soil, showed substantial variability of survival of *Escherichia coli* O157:H7 strains of human or animal origins, data are lacking on the variability in survival of different *Salmonella* strains in soil amended with poultry manure. Additionally, recent studies (Andino and Hanning, 2015; Harrand et al., 2019) have shown that differences between pathogen strains can result in substantially different responses to environmental stresses. As such, the study reported here was performed to (i) investigate survival and die-off between 12 *Salmonella* strains following inoculation in poultry litter amended soil, and (ii) characterize the effect of soil-type (sandy-loam vs. clay-loam), irrigation regimen (daily vs. weekly), and amendment (poultry litter vs. non-amended) on *Salmonella* survival and die-off of a selection of 12 strains.

## Materials and Methods

### Experimental Design

The design of this greenhouse study was adapted from a peer-reviewed guidance document, that outlines how model system studies should be performed to investigate pathogen die-off in preharvest soils following BSAAO (Harris et al., 2013). Since the primary objective of this study was to characterize population dynamics of different *Salmonella* strains following soil amendment with poultry litter, each of the study’s three independent trials consisted of 24 pots that were irrigated daily (12 with sandy-loam soil and 12 with clay loam soil), and inoculated with one of 12 *Salmonella* strains (i.e., one strain per pot). Since past studies (Holley et al., 2006; Semenov et al., 2009; Underthun et al., 2018; Wang et al., 2018), have shown that irrigation regimen and soil moisture can affect *Salmonella* survival, three strains were randomly selected and used to inoculate an additional six pots per trial (three with sandy-loam soil and three with clay-loam soil; one strain per pot), for a weekly irrigation regimen (pots irrigated weekly). To account for the impact of amendment (compared to not amending the soil), one strain was randomly selected and used to inoculate an additional two non-amended pots per trial (one with sandy-loam soil and one with clay-loam soil). In total, the study consisted of 96 pots [(12 amended pots irrigated daily + 3 amended pots irrigated weekly + 1 non-amended pot irrigated daily) × 2 soil-types = 32 pots per trial × 3 independent trials = 96 total pots], with three replicates for each combination of soil-type, amendment, strain, and irrigation regimen. This number of replicates is consistent with several prior studies (Natvig et al., 2002; Semenov et al., 2009; Schwarz et al., 2014; Fongaro et al., 2017; Hruby et al., 2018; Underthun et al., 2018).

Throughout the study greenhouse conditions were maintained to replicate temperature and humidity during the spring growing season in VA, United States. To ensure that greenhouse conditions remained constant, these parameters were continuously monitored and recorded each time samples were collected (HOBO Micro Station Data Loggers, Onset, Bourne, MA, United States; Supplementary Figure S1).

### Inoculum Preparation

The 12 *Salmonella enterica* strains used here were selected to represent a diversity of serovars and sources (environmental, clinical, food, animal; Table 1). Strains were pre-adapted to grow in the presence of 50 μg/ml nalidixic acid (NA) to assist in distinguishing the inoculum strains from background microflora, as described by Parnell et al. (2005). Prior to the start of each trial, each NA-adapted strain was cultured from frozen stock (−80°C) on tryptic soy agar ((TSA) plates (Difco, BD, Sparks, MD, United States) supplemented with 50 μg/ml NA and incubated at 37°C for 24 h. The inoculum was prepared using a modified version of a the protocol described in Sharma et al. (2016). Briefly, three to five colonies per strain were taken from the TAS plates, and inoculated into 200 ml of tryptic soy broth (TSB; Difco, BD) supplemented with 50 μg/ml NA. The broth was then incubated at 37°C for 18 h with agitation (140 rpm). To achieve a target starting inoculum of 6 log CFU/ml for each strain, a 10 ml aliquot of each 18 h culture was added to separate 990 ml of 0.1% peptone water (Difco, BD). To confirm the concentration of each inoculum for each strain, serial dilutions in 0.1% peptone water were plated onto TSA and xylose lysine tergitol (XLT-4) agars (Difco, BD) supplemented with 50 μg/ml NA. Following incubating at 37°C for 24 h, colonies on the TSA and XLT-4 plates were enumerated, and log CFU/ml of *Salmonella* was calculated.

**Table 1:** Description of the 12 *Salmonella* strains including source, and time to last *Salmonella* detection (survival) in poultry litter amended clay-loam and sandy-loam soils by daily and weekly irrigation.

Strain	Source	Time (day) to last detect (*Salmonella* survival)^a^					
		Poultry litter amended				Non-amended	
		Daily irrigation^b^		Weekly irrigation^c^		Daily irrigation	
		Clay-loam	Sandy-loam	Clay-loam	Sandy-loam	Clay-loam	Sandy-loam
*S.* 4,5,12:i:-	Environmental (water)	168	112				
*S.* Braenderup	Food (cucumber)	210	112	252	336		
*S.* Enteritidis	Food (romaine lettuce)	168	112				
*S.* Javiana	Environmental (sediment)	112	210				
*S.* Meleagridis	Animal (cattle)	84	112	210	252		
*S.* Montevideo	Food (iceberg lettuce)	210	112				
*S.* Muenchen	Food (orange juice)	84	84				
*S.* Newport-F	Food (almond)	168	112	336	252		
*S.* Newport-E	Environmental (water)	168	112			56	56
*S.* Paratyphi B	Environmental (sediment)	168	112				
*S.* Poona	Clinical (human)	168	112				
*S.* Saintpaul	Clinical (human)	168	168				

a Values represent the days post-inoculation (dpi) that *Salmonella* was detectable, including by enrichment of a 25 g soil sample.

b Adjusting for the water loss on a daily basis.

c Adjusting for the water loss on a weekly basis.

### Soil and Poultry Litter Collection, and Inoculation

The sandy-loam and clay-loam soil used in the study reported here were obtained from commercial produce fields in Painter, VA, United States and Petersburg, VA, United States, respectively. Soil was collected from the top 20 cm of the field, sieved to homogenize the soil, and remove rocks, debris, and large aggregates, and air-dried for 7 days. Poultry litter was obtained from a chicken operation in Melfa, VA, United States the morning of each trial. Soil and poultry litter physicochemical properties (e.g., pH, moisture, total carbon, total nitrogen, and phosphorus), were determined by an external lab (Waypoint Analytical; Richmond, VA, United States), and are reported in Supplementary Tables S1 and S2.

Immediately prior to the start of each trial, soil was weighed into 1 kg portions and added to sterile 1.89 L plastic pots. Poultry litter was added to soil at a rate of 8.9 Mg/ha (4 tons per acre) to reflect commercial practices. The amended soil was then inoculated with one of the 12 strains. Briefly, 100 ml of each *Salmonella* strain inoculum were mixed with 1 kg of amended soil and sterile distilled water, where it was thoroughly homogenized using a 30 s shake, 30 s rub, 30 s shake procedure, repeated for 5 min. Sterile distilled water was then added to achieve a soil moisture-level of 13 and 23% for sandy-loam and clay-loam soils, respectively. Pre-study experiments to test the inoculation protocol showed a homogenous distribution of *Salmonella* in the amended soil following inoculation (Supplementary Table S3; ~4 log CFU/g.). Following homogenization, the inoculated soil was returned to the pot, and each pot transferred to the greenhouse.

In parallel with each trial, six control pots were also run: un-inoculated amended sandy- and clay-loam soil irrigated daily (one per soil-type) and weekly (one per soil-type), and un-inoculated non-amended sandy- and clay-loam soils irrigated daily (one per soil type). Control samples were prepared and handled identically as inoculated soil.

Throughout the study, sterilized distilled water was added to each pot to bring soil moisture to levels that were typically observed in agricultural fields (13% for sandy-loam and 23% for clay-loam pots). Water was either added (categorized as an “irrigation event”) on a daily or weekly basis to ensure soil moisture was maintained (at +/− 2%) using soil moisture probes (TDR150; Spectrum, Aurora, IL, United States). Irrigation regimens (daily or weekly) and soil moisture levels were selected to cover a variety of crops and practices within the Mid-Atlantic and Southeastern regions following the Mid-Atlantic Commercial Vegetable Production Guide (Wyenandt et al., 2016) and Southeastern US Vegetable Crop Handbook (Kemble et al., 2017).

It is important to note that immediately prior to poultry litter inoculation, the soil and poultry litter were tested for endemic NA-tolerant *Salmonella*. Soil and poultry litter (25 g sample) were tested for *Salmonella* before each trial according to protocols described below (Underthun et al., 2018; Food and Drug Administration, 2019).

### Sampling and *Salmonella* Enumeration

Twenty-five gram composite soil samples were collected from each pot 0, 0.167 (4 h), 1, 2, 4, 7, 10, 14, 21, 28, 56, 84, 112, 168, 210, 252, and 336 days post-inoculation, or until *Salmonella* could no longer be detected after two consecutive sample collections. Following sample collection, *Salmonella* levels were enumerated. To make the composite soil sample, five 5 g samples were collected from each pot, and combined with 225 ml of buffered peptone water (BPW; Difco, BD) in a sterile, filtered Whirl-pak bag (Nasco, Modesto, CA, United States). Each bag was hand massaged for 60 s. Standard direct plating method was used until counts neared the limit of detection (LOD; Underthun et al., 2018; Food and Drug Administration, 2019; LOD = 1 log CFU/g). Briefly, 1 ml of each soil sample homogenate (25 g sample and 225 ml BPW) was serially diluted in 9 ml of 0.1% peptone water, and 0.1 ml was plated on XLT-4 supplemented with 50 μg/ml NA (LOD=). Separately, 1 ml (0.25 ml aliquoted on each of four XLT-4 plates) was plated from the sample bag to reach a LOD of 1 log CFU/g (Flessa et al., 2005). All XLT-4 plates were incubated at 37°C for 24 h.

Once *Salmonella* counts neared the LOD for the direct plating method, a modified *Salmonella* most probable number (MPN) method (Gartley et al., 2018; Jay-Russell et al., 2018a,b; Food and Drug Administration, 2019) was used to increase the LOD to 0.25 (−0.6 log) MPN/g. Briefly, 5 ml of the homogenate was transferred into the first column of a 48 well reservoir (eight by six well reservoir; with subsequent columns filled with 4.5 ml of BPW). Each sample was serially diluted (1:10) six times and incubated at 37°C for 20 h shaking (50 rpm). A 50 uL aliquot from each well was then transferred into the corresponding wells of a 48 well reservoir of Rappaport Vassiliadis (RV) broth (Difco, BD; 5 ml of RV in each well). After incubating at 42°C for 48 h shaking (50 rpm), 10 μl from each well was channel streaked onto XLT-4 supplemented with 50 μg/ml nalidixic acid, and incubated at 37°C for up to 48 h. The number of *Salmonella* positive streaks (out of eight total streaks) was recorded. A presumptive *Salmonella* positives was indicated by black streaks with a pink/yellow halo. Presumptive *Salmonella* positives were re-streaked onto TSA plates, incubated at 37°C for 24 h. Up to four colonies per streak were confirmed as *Salmonella* by PCR amplification of the *invA* gene (McEgan et al., 2013; Strawn et al., 2013a). PCR-confirmed streaks were counted, and MPN values calculated as previously described (http://standards.iso.org/iso/7218/). The remainder of the sample homogenate was enriched concomitantly with the MPN enumeration using standard FDA BAM methods (Food and Drug Administration, 2019; LOD = 1 cell/25 g).

### Statistical Analysis

All analyses were performed in R version 3.3.1 or 3.3.5 (R Foundation for Statistical Computing, Vienna, Austria). For all analyses, the data were divided into three subsets: (i) amended pots irrigated daily (“the daily dataset”; *N* = 72), (ii) amended pots that were inoculated with *S.* Braenderup, *S.* Newport-F, or *S.* Meleagridis, since these were the three strains that received both daily and weekly irrigation (“the irrigation dataset”; *N* = 18), and (iii) pots that were inoculated with *S.* Newport-E, since this was the only strain that was inoculated into both amended and non-amended pots (“the amendment dataset”; *N* = 6).

#### Effect of Soil-Type, Irrigation, and Amendment on *Salmonella* Survival and Die-off

The daily dataset was used to quantify the impact of soil-type on die-off rate, while controlling for strain. The irrigation dataset was used to quantify the impact of irrigation regimen on die-off rate, while controlling for strain and the amendment dataset was used to quantify the impact of amendment on die-off rate, while controlling for strain. It is important to note that the small size of the irrigation and amendment datasets is a major limitation of this study; however, these analyses were included in the study reported here for comparison to prior literature (Holley et al., 2006; You et al., 2006; Semenov et al., 2009; Sharma et al., 2016; Underthun et al., 2018; Wang et al., 2018; Shah et al., 2019), while investigating strain differences. To perform these analyses, two sets of general linear models were developed. The outcome of the models were either (i) time to last detection of *Salmonella* (i.e., the last time point where *Salmonella* could be isolated from a sample, including 25 g enrichments), which was used to examine the effect of each soil-type, strain, irrigation regimen, and soil amendment on *Salmonella* survival and (ii) concentration of *Salmonella* at each time point (log CFU or MPN/g). Depending on the dataset, a fixed effect of irrigation regimen (weekly or daily; irrigation dataset) or amendment (poultry litter-amended or non-amended; amendment dataset) was also included. In models where the outcome was *Salmonella* concentration, time since inoculation was also included as a fixed effect. The effect for time was interpreted as the average daily die-off rate (i.e., average change in log CFU or MPN/g per day). All general linear mixed models included soil-type (sandy- or clay-loam) and trial (1, 2, or 3) as fixed effects, and strain as a random effect.

#### Effect of Strain on *Salmonella* Survival and Die-off

The daily dataset was used to compare the die-off rate for each of the 12 strains in poultry litter amended soils. Using the LmList function in the *lme4* R package separate linear models were built for each *Salmonella* strain. The outcome was *Salmonella* concentration, and the fixed effects were time since inoculation, trial, and soil-type. Die-off rates between strains were compared visually using the 95% confidence intervals (CI) for die-off rate (i.e., the effect estimate for time since inoculation). If the 95% CI did not overlap, it was concluded that the die-off rates between the two strains were significantly different. A limitation to these analyses is the small number of pots inoculated with each strain; however, prior studies have used similar study units (Natvig et al., 2002; Semenov et al., 2009; Schwarz et al., 2014; Fongaro et al., 2017; Hruby et al., 2018; Underthun et al., 2018).

#### Effect of Interactions Between Soil-Type, Strain Irrigation, and Amendment on *Salmonella* Survival

To identify specific combinations of soil-type, irrigation regimen, soil amendment, and strain associated with decreased or increased *Salmonella* survival, a conditional inference tree (cTree) was built using the full dataset and the partykit package. Since the smallest number of pots included in a given treatment were 3, the minbucket parameter was set to 6 to prevent overfitting; the mincriterion parameter was also set to 0.95 to prevent overfitting. Separate trees were also built using the same parameters, but using the individual datasets (as opposed to the full dataset), to see if there were additional interactions, not evident when the full dataset was used.

## Results and Discussion

**Figure 1:**
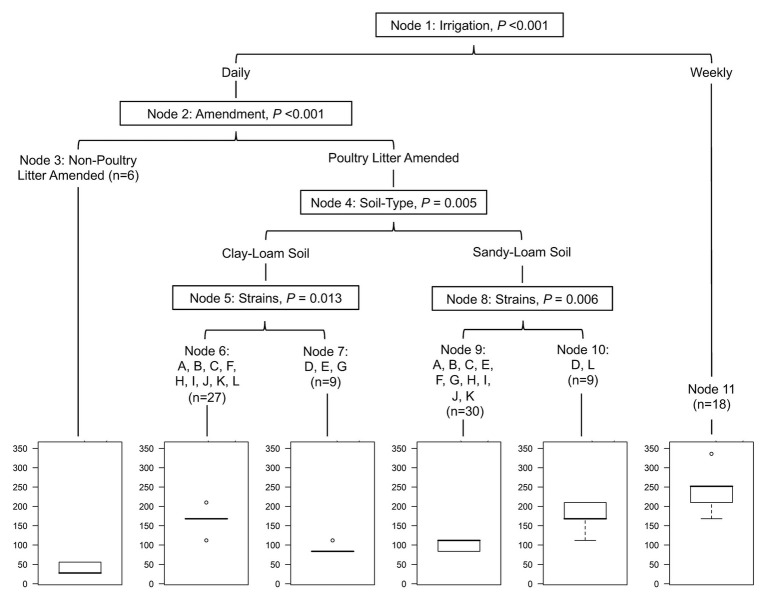
Conditional inference tree (cTree) built showing potential interactions in the full dataset between irrigation regimen, soil amendment, soil-type, and strain, and their impact on the total length of time *Salmonella* remained detectable in the study reported here. For example, *Salmonella* survives the longest in pots receiving weekly irrigation, and the shortest in pots receiving no soil amendment. However, for amended pots receiving daily irrigation, survival time was dependent on soil-type and strain. Bar plots show the expected survival of *Salmonella* (d) in each terminal node. Letters represent the following *Salmonella* strains: A (*S.* 4,5,12:i:-), B (*S.* Braenderup), C (*S.* Enteritidis), D (*S.* Javiana), E (*S.* Meleagridis), F (*S.* Montevideo), G (*S.* Muenchen), H (*S.* Newport-F), I (*S.* Newport-E), J (*S.* Paratyphi B), K (*S.* Poona), and L (*S.* Saintpaul).

The study reported here characterized strain variability and the influences of soil-type, irrigation regimen, and amendment on the concentration of *Salmonella*. The regression tree analysis showed that strain, soil-type, irrigation regimen, and amendment all significantly influenced the survival of *Salmonella* in agricultural soils (Figure 1). The findings of the regression tree analysis are supported by the results of the general linear mixed models, which also showed that soil-type, irrigation regimen, and amendment significantly impacted *Salmonella* survival time (Table 2); as well as, die-off rate (Table 3; *p* ≤ 0.05). To our knowledge, this is the first study to quantify the variability for 12 *Salmonella* strains in poultry litter amended soils under different conditions (i.e., soil-types) and management practices (i.e., irrigation regimens). In fact, nearly 40 years ago, Zibilske and Weaver (1978) described a critique of their *Salmonella* survival in soil study that only a single strain was used, as such the study reported here is long overdue. There are some limitations to the study reported here, such as the sample size, and the fact that this was a greenhouse study that cannot precisely replicate field conditions. As such, additional studies with a larger number of samples are needed to determine if these findings are reproducible; as well as test other factors such as, different soil-types (e.g., true sand and loam), irrigation practices (e.g., flood and dry-land production), and soil amendments (e.g., bovine and horse). Despite these limitations, this study is consistent with past research (using single strains, or cocktail; You et al., 2006; Semenov et al., 2009; Andino and Hanning, 2015; Underthun et al., 2018; Harrand et al., 2019; Shah et al., 2019), and also shows the variability of *Salmonella* strains under different agricultural parameters. Data and findings generated here can be used in (i) future risk assessments, (ii) selection of strains for future challenge and validation studies, especially when used together with whole genome sequencing data that may characterize the molecular determinants for environmental adaptation and survival (e.g., worst case contamination scenarios), and (iii) identification of best practices for application of BSAAO, like poultry litter, to agricultural fields.

**Table 2:** Results of general mixed models that were developed to investigate the impact of soil-type, irrigation regimen, and soil amendment on *Salmonella* survival (i.e., total days *Salmonella* was at detectable levels for each pot in this study).

Data set (R^2^)	Factor	Effect estimate	95% CI	*p*-value
Soil-type (0.35)^a^	Trial (one = reference)^b^			0.188
	Two	−29.56	−19.53, 17.20	0.902
	Three	−15.75	−34.11, 2.61	0.101
	Sandy soil (clay = reference)^d^	−29.56	−44.55, −14.56	<0.001
Irrigation (0.73)^c^	Trial (one = reference)^b^			0.580
	Two	−12.83	−45.37, 19.71	0.462
	Three	−17.50	−50.04, 15.04	0.317
	Sandy soil (clay = reference)^d^	−10.89	−37.46, 15.68	0.444
	Weekly irrigation regimen (daily = Reference)^e^	127.56^e^	101.00, 154.12	<0.001
Amendment (0.86)^f^	Trial (one = reference)^b^			0.109
	Two	14.00	−19.50, 47.50	
	Three	−21.00	−54.50, 12.50	
	Sandy soil (clay = reference)^d^	−23.33	−50.68, 4.02	0.083
	Poultry litter amendment (no amendment = reference)	88.67^g^	61.32, 116.02	<0.001

a 17% of variance in *Salmonella* survival is explained by the fixed effects shown here, and 18% of variance in *Salmonella* survival is explained by the random effect of strain.

b The estimated change in time to last detect when the samples were collected as part of trials two or three compared to trail one.

c 65% of variance in *Salmonella* survival is explained by the fixed effects shown here, and 8% of variance in *Salmonella* survival is explained by the random effect of strain.

d The estimated change in time (days) to last detect when the soil was sandy-loam compared to clay-loam (which was the reference-level). For example, this means that *Salmonella* is expected to survive 30 days longer in clay soil than sandy soil based on the soil-type model.

e *Salmonella* was predicted to survive 128 days longer when weekly compared to daily irrigation was performed.

f 86% of variance in *Salmonella* survival is explained by the fixed effects shown here since there were no random effects used in the amendment model.

g *Salmonella* was predicted to survive 89 days longer in poultry litter amended pots compared to non-amended pots.

**Table 3:** Results of log-linear general mixed models that were developed to investigate the impact of soil-type, irrigation regimen, and soil amendment on *Salmonella* daily die-off rate.

Data set (R^2^)	Factor	Effect estimate	95% CI	*P*-value
Soil-type (0.70)^a^	Daily die-off rate^b^	−0.03	−0.03, −0.03	<0.001
	Trial (one =reference)^c^			0.004
	Two	−0.19	−0.34, −0.04	0.015
	Three	−0.25	−0.40, −0.10	0.001
	Sandy soil (clay = reference)^e^	0.66	0.54, 0.79	<0.001
Irrigation (0.71)^d^	Daily die-off rate^b^	−0.02	−0.02, −0.02	<0.001
	Trial (one = reference)^c^			0.034
	Two	−0.24	−0.48, 0.00	0.051
	Three	−0.30	−0.55, −0.06	0.014
	Sandy soil (clay = reference)^e^	0.43	0.24, 0.63	<0.001
	Irrigation regimen (daily = reference)^f^	−0.03	−0.23, 0.18	0.808
Amendment (0.77)^g^	Daily die-off rate^b^	−0.05	−0.05, −0.04	<0.001
	Trial (one = reference)^c^			0.468
	Two	−0.19	−0.58, 0.21	0.356
	Three	−0.20	−0.60, 0.20	0.331
	Sandy soil (clay = reference)^e^	0.82	0.50, 1.15	<0.001
	Poultry litter amendment (no amendment = reference)^h^	2.60	2.25, 2.94	<0.001

a 61% of variance in *Salmonella* die-off is explained by the fixed effects shown here, and 9% of variance in *Salmonella* die-off is explained by the random effect of strain.

b The die-off rate represents the daily decrease in log_10_ *Salmonella* concentration when all other variables in the model were held constant.

c The estimated difference in log_10_ *Salmonella* concentration when the samples were collected as part of trials two or three compared to trail one.

d 52% of variance in *Salmonella* die-off is explained by the fixed effects shown here, and 19% of variance in *Salmonella* die-off is explained by the random effect of strain.

e The estimated difference in log_10_ *Salmonella* concentration when the soil was sandy-loam compared to clay-loam (which was the reference-level). For example, this means that the log_10_ *Salmonella* concentration is expected to be 0.66 log_10_ most probable number (MPN) or CFU higher in sandy-loam soil than clay-loam soil based on the soil-type model.

f The log_10_ *Salmonella* concentration is expected to be 0.03 log_10_ MPN or CFU lower when irrigation was performed weekly as opposed to daily.

g 77% of variance in *Salmonella* survival is explained by the fixed effects shown here since there were no random effects used in the amendment model.

h The log_10_ *Salmonella* concentration is expected to be 2.60 log_10_ MPN or CFU higher when the soil was amended compared to non-amended.

### Survival and Die-off Rates in Amended Soils Receiving Daily Irrigation Were Significantly Associated With *Salmonella* Strain

**Figure 2:**
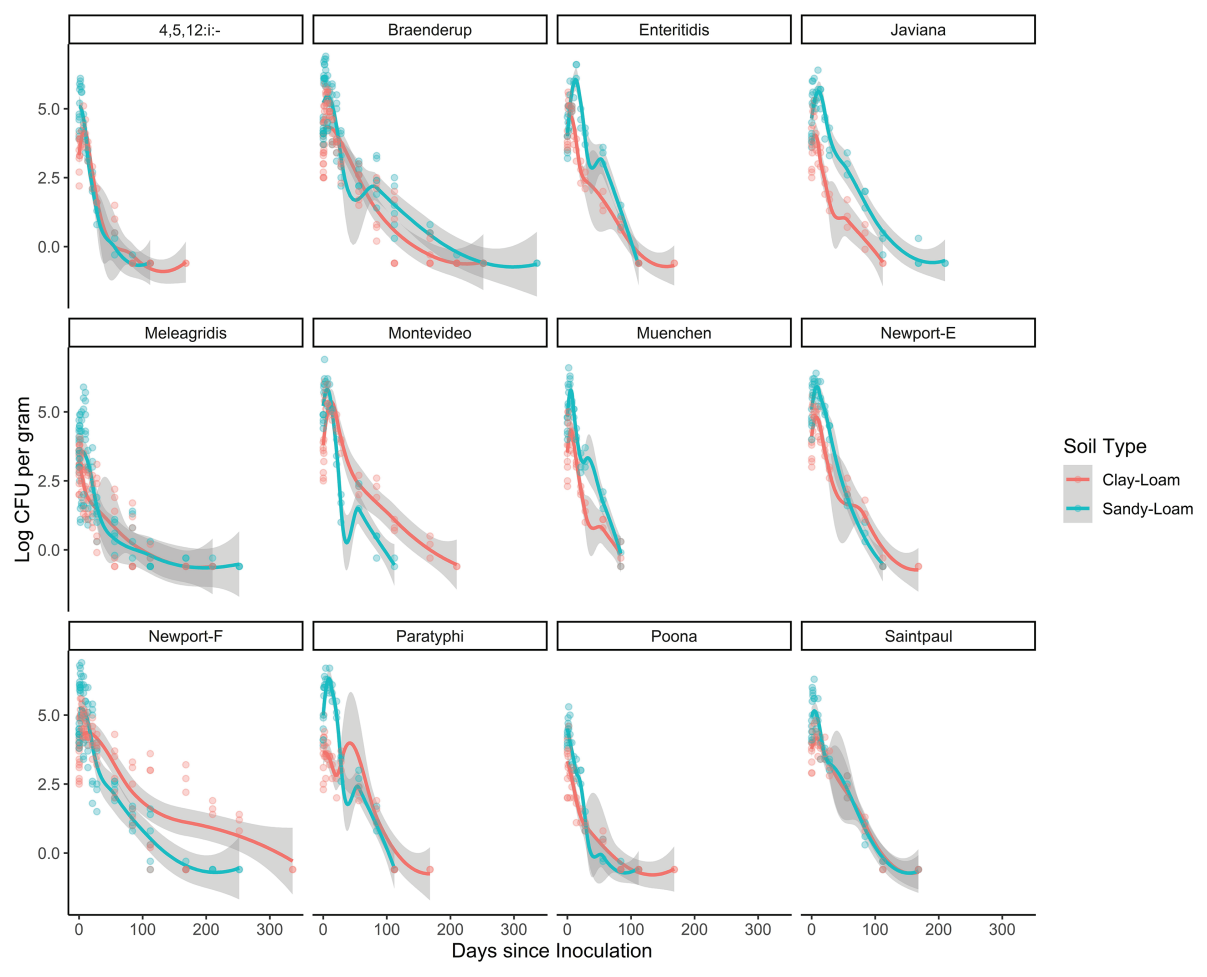
Loess-smoothed regressions showing concentration and 95% confidence intervals (gray shading) for each strain in poultry litter-amended clay-loam (red line) and sandy-loam (teal line) soils.

Across the study, the total time that *Salmonella* survived ranged between 84 and 210 days (mean = 144 days; Figure 2). In poultry litter amended sandy-loam soil pots, the concentration of all strains, except *S.* Poona, increased between inoculation and 7 days post-inoculation (dpi), and remained elevated until 14 dpi (Supplementary Table S4). On average, *Salmonella* concentrations started to decrease 14 dpi (Supplementary Table S4). Conversely, in amended clay-loam soil, all strains observed a 0.5–1.5 log CFU or MPN/g reduction 4 h dpi; for some of the strains, subsequent growth occurred with an increase between inoculation and 4–7 dpi (Supplementary Table S5). While *S.* Saintpaul was the only strain to sort into the node for longer survival (average 168 days) in both sandy- and clay-loam amended soils, *S.* Javiana, and *S.* 4,5,12:i:-, *S.* Braenderup, *S.* Enteritidis, *S.* Montevideo, *S.* Newport-F, *S.* Newport-E, *S.* Paratyphi B, and *S.* Poona (cTree) were sorted in to the longer survival nodes for sandy- and clay-loam soils, respectively (Figure 1). This suggests that the effect of soil-type, and other factors, on survival may be strain-specific; however, investigating potential interactions between strain, soil-type, and the other factors included here were outside the scope of the study and should be investigated as part of future research projects.

In the general linear models, strain accounted for up to 18% of variance in survival and 9% of variance in die-off rate, suggesting variation between strains (Tables 2 and 3). Separately from the general linear models’ individual log-linear models were developed to compare die-off rates (i.e., the effect estimate for time) between *Salmonella* strains (Table 4). By comparing the 95% CI for the die-off rates, we could identify significant differences between strains. For example, *S.* Muenchen had a faster daily die-off rate (−0.060 log CFU or MPN/g per day; standard error = 0.005), compared to all 12 strains. Conversely, *S.* Braenderup, *S.* Meleagridis, and *S.* Newport-F had significantly slower daily die-off rates, compared to the other nine strains (Table 4). *S.* Javiana and *S.* Montevideo had slower die-off rates, than *S.* Muenchen, but had faster die-off rates than *S.* Braenderup, *S.* Meleagridis, and *S.* Newport-F (Table 4). Interestingly, there was a significant effect of soil-type for nine of the 12 strains, with *Salmonella* concentrations being higher in sandy-loam soils, compared to clay-loam soils (Table 4). This supports the conclusion described above that the effect of environmental factors, such as soil-type, on *Salmonella* survival and die-off is strain-specific; however, it may also be a function of the small sample size here.

**Table 4:** Results of linear models that were developed to compare die-off rate between each of the 12 *Salmonella* strains used in poultry litter amended soil over time by log linear models.

	Soil-type (reference = clay soil)			Daily die-off rate^a^			Trial 2 (reference = trial 1)			Trial 3 (reference = trial 2)			R^2^
Serovar	Effect^b^ estimate	SE	*p*-value	Effect^c^ estimate	SE	*p*-value	Effect^d^ estimate	SE	*p*-value	Effect^d^ estimate	SE	*p*-value	
4,5,12:i:-	0.32	0.23	0.167	−0.04	0.003	<0.001	−0.04	0.28	0.893	−0.20	0.28	0.463	0.74
Braenderup	0.82	0.15	<0.001	−0.02	0.001	<0.001	−0.22	0.18	0.273	−0.31	0.19	0.097	0.76
Enteritidis	0.68	0.23	0.003	−0.04	0.003	<0.001	−0.05	0.28	0.849	−0.13	0.28	0.653	0.79
Javiana	1.55	0.23	<0.001	−0.03	0.002	<0.001	−0.08	0.27	0.778	−0.19	0.27	0.495	0.83
Meleagridis	0.55	0.16	<0.001	−0.02	0.001	<0.001	−0.19	0.19	0.336	−0.33	0.19	0.091	0.55
Montevideo	−0.08	0.23	0.730	−0.03	0.002	<0.001	−0.31	0.27	0.265	−0.37	0.27	0.178	0.70
Muenchen	1.19	0.24	<0.001	−0.06	0.005	<0.001	−0.27	0.29	0.355	−0.07	0.29	0.809	0.83
Newport F	−0.05	0.15	0.738	−0.02	0.001	<0.001	−0.32	0.19	0.084	−0.30	0.19	0.111	0.63
Newport E	0.70	0.23	0.002	−0.04	0.003	<0.001	−0.21	0.28	0.453	−0.35	0.28	0.220	0.84
Paratyphi	1.47	0.23	<0.001	−0.04	0.003	<0.001	−0.04	0.28	0.877	−0.12	0.28	0.660	0.80
Poona	0.64	0.23	0.006	−0.04	0.003	<0.001	−0.18	0.28	0.519	−0.16	0.28	0.562	0.73
Saintpaul	0.53	0.22	0.017	−0.04	0.002	<0.001	−0.33	0.27	0.232	−0.36	0.27	0.194	0.87

a The die-off rate represents the daily decrease in log_10_ *Salmonella* concentration when all other variables in the model were held constant.

b The estimated change in log10 Salmonella concentration for the given strain when the soil was sandy-loam compared to clay-loam (which was the reference-level). For example, this means that the log10 Salmonella Braenderup concentration is expected to be 0.82 log10 MPN or CFU higher per gram for pots with sandy-loam compared to clay-loam soil.

c The estimated change in log_10_ *Salmonella* concentration for the given strain for each 1 day increase in time since inoculation. For example, this means that the log_10_ *Salmonella* Braenderup concentration is expected to be decreased by 0.02 log_10_ MPN or CFU higher per gram for each day since inoculation.

d The estimated difference in log10 *Salmonella* concentration for the given strain for pots included in trials 2 and 3 compared to trial 1.

This study examined the variation in the survival and die-off of a large number of *Salmonella* strains (*n* = 12) in amended sandy- and clay-loam soils. The comparison of individual (single) strains (not in cocktails) is important, as recent papers (Andino and Hanning, 2015; Harrand et al., 2019) have shown that different *Salmonella* strains can vary substantially in their responses to environmental stresses (e.g., temperature and moisture), which may impact survival. Multiple studies have investigated the survival of a single strain (e.g., You et al., 2006; Semenov et al., 2009; Shah et al., 2019) or a strain cocktail (e.g., Underthun et al., 2018) in amended and non-amended soils. For example, You et al. (2006) reported the die-off rate for *S.* Newport in soil amended with bovine manure similar to those reported here (ranged from −0.03 to −0.04 log CFU/g per day). That being said, the current study does not observe the interactions between different *Salmonella* strains and its influence on the survival in soil and poultry litter amended soil. Further studies may look to investigate the interactions of different *Salmonella* strains in combination.

### *Salmonella* Survival and Die-off Rates Were Significantly Different in Poultry Litter Amended Soils That Received Weekly Irrigation, Compared to Daily Irrigation

**Figure 3:**
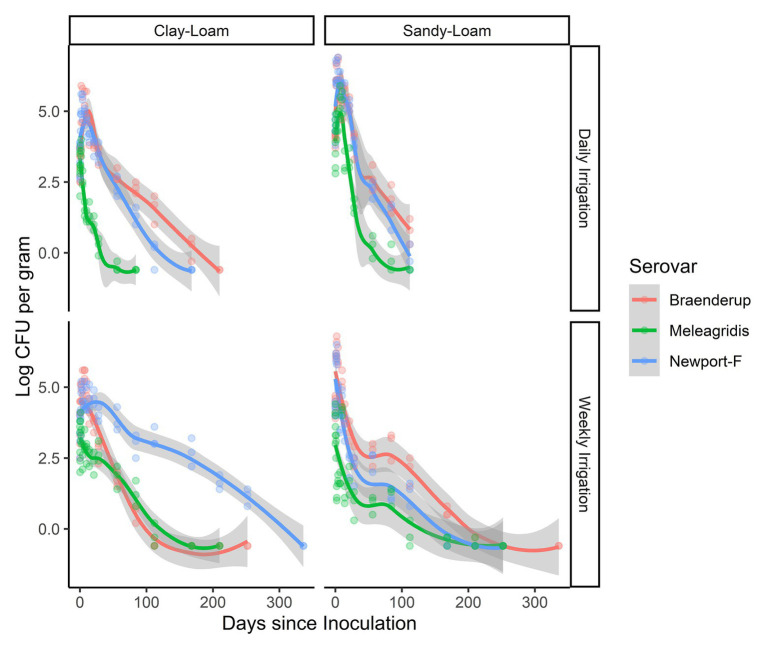
Less-smoothed regressions showing concentration and 95% confidence interval (gray shading) for each strain *S.* Braenderup (red line), *S.* Meleagridis (green line), and *S.* Newport-F (blue line) in poultry litter-amended clay and sandy soils.

Three strains (*S.* Braenderup, *S.* Newport-F, and *S.* Meleagridis) were exposed to two different irrigation regimens (daily and weekly). *Salmonella* survived on average 128 days longer (95% CI 101.00, 154.12) when irrigated weekly compared to daily according to the general linear model (Table 2). While, *Salmonella* concentration was, on average, higher in poultry litter amended soils that received daily irrigation, compared to weekly irrigation (Figure 3), this difference was not significant with regard to die-off rate (Table 3). The magnitude of the effect of irrigation regimen on survival and die-off was almost 10-times that of soil type (Tables 2 and 3). Given our finding that die-off rates varied significantly between strains, and total survival time appeared to be dependent on both strain and soil-type in the regression tree, future studies should test if the effect of irrigation regimens substantially influence other *Salmonella* strain’s survival. Additionally, irrigation regimen may also interact with other soil characteristics (e.g., soil particle size and slope) to influence *Salmonella* survival (Sharma and Reynnells, 2018); thus, future studies should investigate the effect of such interactions.

### *Salmonella* Survived Significantly Longer in Poultry Litter Amended Soils Compared to Non-amended Soils

**Figure 4:**
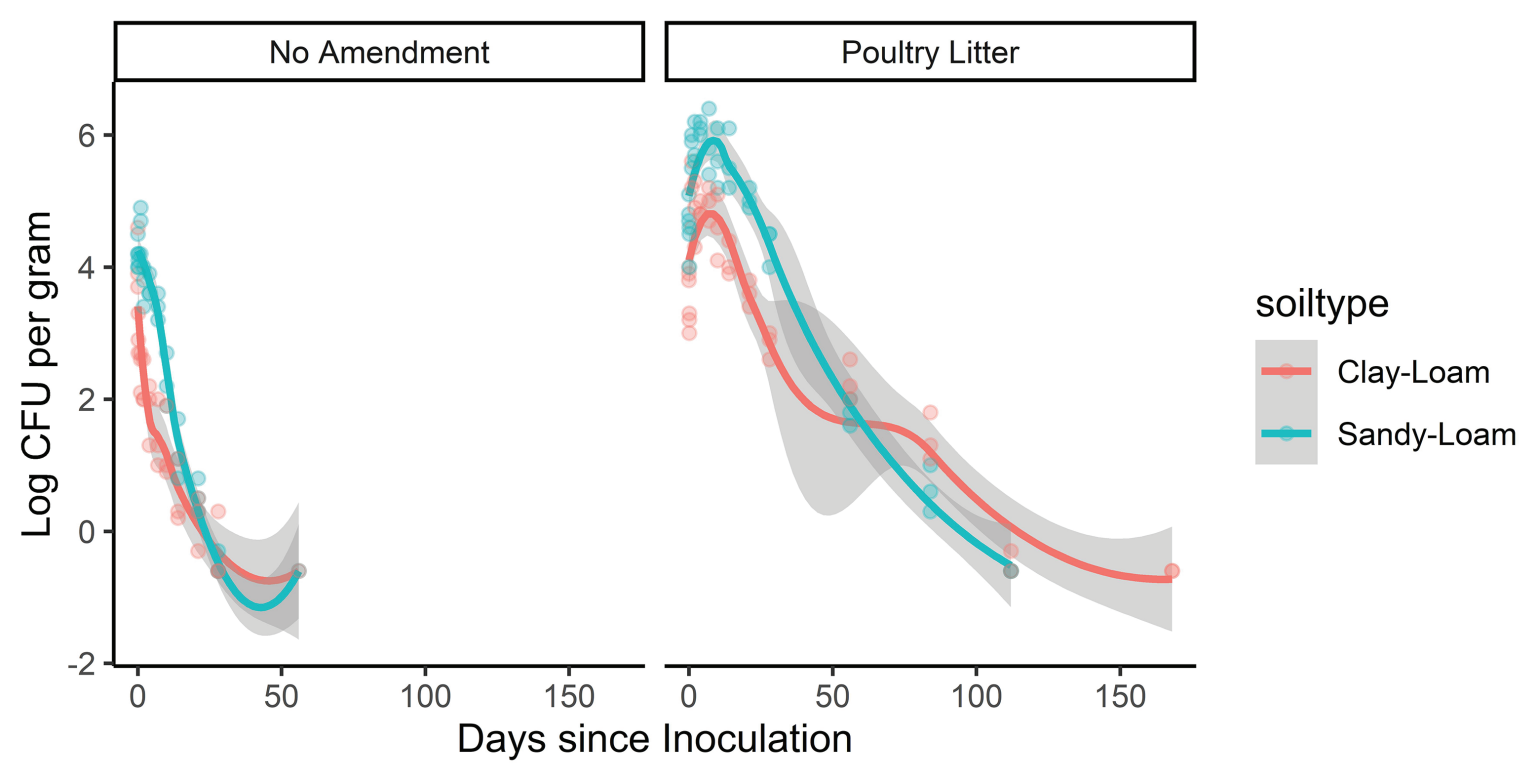
Loess-smoothed regressions showing concentration and 95% confidence intervals (gray shading) for *S.* Newport-E in poultry litter-amended clay-loam (red line) and sandy-loam (teal line) soils.

Amendment with poultry litter significantly prolonged *Salmonella* Newport-E survival by 89 days (95% CI = 61.32, 116.02, *p* ≤ 0.001; Table 2; Figure 4). Overall, these findings are consistent with previous studies (Baloda et al., 2001; Islam et al., 2004a; Holley et al., 2006; You et al., 2006; Nyberg et al., 2010; Ongeng et al., 2011; Hruby et al., 2018; Shah et al., 2019) that reported *Salmonella* survival increased in soils amended with BSAAO (e.g., poultry litter and bovine manures). For example, You et al. (2006) found that addition of bovine manure to agricultural soils increased *S.* Newport concentrations by as much as 400% within the first 3 days. Baloda et al. (2001) observed that *Salmonella* survive up to 299 days in hog slurry amended agricultural soils, while Hruby et al. (2018) detected *Salmonella* in soil samples from poultry litter amended plots nearly a full year after application. Additionally, the die-off rate of *S.* Newport-E was slower in poultry litter amended soils, compared to non-amended soils (Table 4). In fact, application of poultry litter amendment increased the concentration of *Salmonella* by 2.6 log CFU/g (95% CI = 2.3, 2.9; *p* < 0.001), which was the largest effect observed for any of the factors (e.g., soil-type, irrigation regimen, and amendment) investigated in the study reported here (Table 3). The study reported here confirms that application of BSAAO to soils (e.g., poultry litter) facilitates *Salmonella* survival. The 120/90 days application interval outlined by the USDA National Organic Program (USDA, 2015, 2020) for growers who use raw manures in produce production is supported by most of the *Salmonella* strains examined in this study, in that *Salmonella* was last detected in a time period shorter than 120 days for 10 of 12 strains in poultry litter amended sandy-loam and for three out of 12 strains in poultry litter amended clay-loam amended soils that received daily irrigation. However, there were certain strains that survived >120 dpi under daily and weekly irrigation regimens; in fact, *S.* Braenderup and *S.* Newport-F survived up to 336 dpi in sandy-loam and clay-loam soil, under a weekly irrigation regimen. This finding suggests that, depending on the target risk reduction or risk associated with consumption of produce grown in amended soil, the USDA National Organic Program may be sufficient for managing food safety risks associated with BSAAO use under certain conditions, but not all conditions. As such, research into condition-specific modifiers may be needed to better manage these risks due to BSAAO application.

### *Salmonella* Strain Die-off Rates Were Significantly Associated With Soil-Type in Poultry Litter Amended and Non-amended Soils Under Both Irrigation Regimens

Six general log-linear models were developed to quantify the effect of soil-type on *Salmonella* survival (*N* = 3 models) and die-off (*N* = 3 models) in (i) amended pots receiving daily irrigation, (ii) amended pots receiving daily vs. weekly irrigation, and (iii) amended vs. non-amended pots receiving daily irrigation (Tables 2 and 3). According to all three die-off models, soil-type was significantly associated with *Salmonella* die-off (*p* < 0.001). In fact, the models for amended pots receiving daily irrigation fixed effects accounted for 17 and 61% of variance in survival and die-off rate, respectively; since the only fixed effects were trial and soil-type, and only soil-type was significant, we can conclude that the majority of the variance was accounted for by soil-type. However, soil-type was only associated with survival in the soil type dataset; this may have been a product of the small number of samples available for use in the irrigation (*N* = 18) and amendment (*N* = 6) datasets. Based on the general linear models, *Salmonella* die-off was slower in clay-loam soils for some of the strains, such as Montevideo and Newport F, compared to sandy-loam soils, which is consistent with past findings. For example, Jechalke et al. (2019) found that *Salmonella* die-off rates were significantly higher in the sand (−0.076 ± 0.014 log CFU/day), compared to loam (−0.034 ± 0.015 log CFU/day). Other studies (Platz, 1980; Bech et al., 2010; Fongaro et al., 2017; Underthun et al., 2018) have demonstrated that soil-type impacts *Salmonella* persistence; in addition to other factors, such as temperature and moisture. For example, one study (Platz, 1980) observed higher isolation rates of *Salmonella* in loam-type and clay-type soils, compared to sandy-type soils. Sandy-type soils typically drain faster with more flow; and thereby, filter microorganisms at a more frequent pace (less-absorptive), compared to loam- and clay-type soils. However, another study (Fongaro et al., 2017) found *Salmonella* survival was significantly lower in clay soil, than in sandy soil. The authors of the study hypothesized clay soils may have more alkaline pH, higher organic matter, and higher nutrient contents (due to reduced leaching and better water retention), which may have influenced survival. Thus, the data reported here, and by others, suggests that soil-type can influence *Salmonella* die-off, and in some cases, *Salmonella* survival. These results suggest future studies should investigate additional soil-types (i.e., different mixtures of sand, loam, and clay) to determine *Salmonella* survival under different field conditions.

## Conclusion

Findings from this study suggest a one-size fits all standard for application of BSAAO to agricultural fields would be challenging as there is significant variability in *Salmonella* survival and die-off by strain; as well as variability by environmental and management factors. Moreover, these findings suggest possible worst-case contamination scenarios that can be used to implement robust recommendations and potential policy decisions. Future studies should critically evaluate strains, soil-type, and management practices to validate potential interventions, guidance, and policy decisions. Based on the results of the study reported here, there are several candidate *Salmonella* strains to use in challenge and validation studies as worst-case contamination scenarios. Prior literature (Harris et al., 2013; Andino and Hanning, 2015; Harrand et al., 2019; Ramos et al., 2019) has described the importance of using worst-case contamination scenarios (i.e., conservative approach) and a diverse set of strains, as survival and behavior can vary considerably by factor (e.g., soil-type). For example, *S.* Saintpaul survived 168 days in sandy- and clay-loam amended soils exposed to daily irrigation, and according to the cTree model had one of the longest times to detect *Salmonella*.

Additionally, *S.* Braenderup, *S.* Meleagridis, and *S.* Newport-F each had significantly slower die-off rates, compared to the other nine strains, with *S.* Newport-F being the slowest of those three influenced by soil-type according to models. Additionally, the use of geographically relevant strains should also be considered to assess persistence and quantify risk of long-term soil contamination, as other studies (Gorski et al., 2011; Strawn et al., 2014; Bell et al., 2015; Jokinen et al., 2015) have demonstrated different *Salmonella* strains can be geographically distributed in specific produce growing regions. For example, *S.* Newport-E (used in the study reported here) is geographically relevant to the Eastern Shore of Virginia, which has sandy-loam soil (also used in this study), and has been associated with tomato-borne outbreaks (Greene et al., 2008; Bell et al., 2015). Overall, *Salmonella* survival and die-off is affected by soil characteristics (e.g., soil-type) and management practices (e.g., irrigation regimen and amendment), and the effect of these factors may be strain-specific. Thus, data can assist in risk assessment and strain selection for use in challenge and validation studies.

## Data Availability Statement

The raw data supporting the conclusions of this article will be made available by the corresponding author.

## Author Contributions

LS, DO, YC, and DI contributed to the study conception and design. LS, SR, and CB contributed to the acquisition of the data. LS, DW, and CB contributed to the analysis and interpretation of the data and drafting of the manuscript. LS, DW, CB, SR, DO, YC, and DI contributed to the critical revisions of the manuscript. All authors contributed to the article and approved the submitted version.

### Conflict of Interest

The authors declare that the research was conducted in the absence of any commercial or financial relationships that could be construed as a potential conflict of interest.
