# Supplementary material for: Strain, Soil-Type, Irrigation Regimen, and Poultry Litter Influence Salmonella Survival and Die-off in Agricultural Soils
Source: Front Microbiol. 2021 Mar 16;12:590303. doi: 10.3389/fmicb.2021.590303 (PMC8007860; doi:10.3389/fmicb.2021.590303)
Supplement: Supplementary file 1 [file Data_Sheet_1.pdf]

Supplemental Materials

Supplemental Figure S1. Box plots for the temperature (C°; A) and relative humidity (%; B) for each of the three independent trials

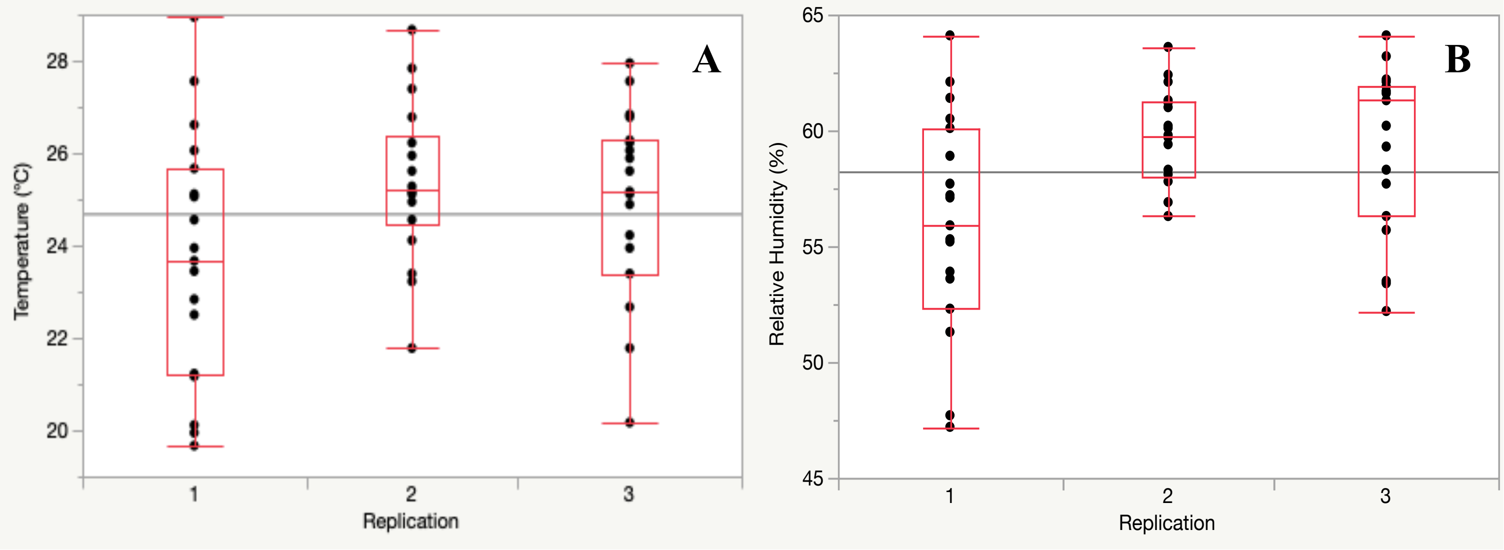

Supplemental Table S1. Physical and Chemical Characteristics of the sandy-loam (SL) and clay-loam (CL) soils[a] used in all experiments

Characteristic	Sandy	Clay
pH	5.7	4.6
Moisture (%)	13	23
Total Organic Carbon (%)[b]	0.5	1.5
Total Nitrogen (mg/kg)[c]	410	1200
Total Kjeldahl Nitrogen (mg/kg)[d]	400	1180
OM (%)	1.0	2.5
Sand (%)	66	41
Silt (%)	24	16
Clay (%)	10	43

[a] SL soil was obtained from a farm on the Eastern Shore of Virginia (Painter, VA, USA), while CL soil was obtained from a farm on mainland Virginia (Petersburg, VA, USA).
[b] Total organic carbon was measured using wet-dry combustion methods and was calculated as loss on ignition (Nelson and Sommers, 2018).
[c] Total nitrogen was measured based on methods described in Mulvaney (Mulvaney, 2018) and Rice et al. (Rice et al., 2017).
[d] Total Kjeldahl nitrogen was determined according to standard method part 4500 N(org) C. Semi-Micro-Kjeldahl methods (Rice et al., 2017).

Supplemental Table S2. Physical and Chemical Characteristics of the PL[a] amendment used in all experiments

Characteristic	Poultry Litter (PL)
pH	8.1
Moisture (%)[b]	25
Phosphorus (%)[c]	1.8
Total Nitrogen (mg/kg)[d]	3,500
Ammonium nitrogen (NH_4-N) (mg/kg)[e]	390

[a] Poultry litter (PL) was obtained from a local chicken operation on the Eastern Shore of Virginia (Melfa, VA, USA).
[b] Moisture measured according to Hoskins et al (Peters et al., 2003).
[c] Phosphorous was determined by EPA Method 3015A (US EPA, 2019).
[d] Total nitrogen was determined by combustion (Dumas method) (Rice et al., 2017).
[e] Ammoniacal nitrogen was measured using flow injection analysis of water extracts (Rice et al., 2017).

Supplemental Table S3. Pre-study experiments to test the inoculation protocol[a] had a homogenous distribution of *Salmonella* in the amended soil following inoculation (sampled 25 g batches; 625 soil was removed from pots)

Batch of soil (25 g)	*Salmonella* concentration (log CFU/g)					
	Test 1	Test 2	Test 3	Test 4	Test 5	Average+/-Standard Deviation
1	4.1	3.7	3.8	4.3	3.9	4.0±0.2
2	3.6	4.1	3.9	3.9	4.0	3.9±0.2
3	4.1	3.9	4.0	3.8	4.0	4.0±0.1
4	4.2	3.9	3.8	4.3	4.1	4.1±0.2
5	3.9	4.0	3.9	4.3	4.0	4.0±0.2
6	4.1	3.9	4.3	4.0	4.0	4.1±0.2
7	3.6	3.8	3.9	4.0	4.1	3.9±0.2
8	4.3	3.8	4.1	4.2	3.9	4.1±0.2
9	3.9	3.8	4.2	4.3	4.2	4.1±0.2
10	3.8	3.6	3.8	4.0	3.9	3.8±0.1
11	4.4	4.1	4.2	4.0	4.2	4.2±0.1
12	3.7	4.1	3.9	3.9	4.1	3.9±0.2
13	4.4	3.8	3.8	4.4	4.0	4.1±0.3
14	4.4	4.0	4.0	4.0	4.0	4.1±0.2
15	4.0	3.8	4.1	4.3	4.0	4.0±0.2
16	4.4	4.1	4.0	4.1	4.0	4.1±0.2
17	3.6	3.7	3.8	4.4	4.2	3.9±0.3
18	4.2	3.8	4.0	3.9	4.1	4.0±0.2
19	4.4	4.0	4.0	4.0	4.1	4.1±0.2
20	3.7	3.6	3.9	4.4	4.1	3.9±0.3
21	4.0	4.0	3.9	4.4	4.1	4.1±0.2
22	4.4	3.7	3.8	3.9	4.1	4.0±0.3
23	4.4	3.7	3.7	4.2	4.1	4.0±0.3
24	3.8	3.9	4.2	4.0	3.9	4.0±0.2
25	4.0	4.0	4.2	3.8	3.9	4.0±0.1
Average +/- Standard Deviation	4.1±0.3	3.9±0.2	4.0±0.2	4.1±0.2	4.0±0.1	

[a] Inoculation protocol described in methods and materials section

Supplemental Table S4. Salmonella concentration (log CFU or MPN/g) by time point for each *Salmonella* strain in poultry-littler amended sandy-loam soils[a]

Time (Day)	4,12:i-	Branderup	Enteritidis	Javiana	Meleagridis	Montevideo	Muenchen	Newport E	Newport F	Paratyphi	Poona	Saintpaul
0	4.2±0.2	4.1±0.1	4.2±0.2	3.8±0.1	4.3±0.2	4.7±0.2	4.7±0.1	4.9±0.1	4.6±0.2	4.2±0.2	4.0±0.1	4.5±0.2
0.17	4.6±0.2	4.0±0.2	3.4±0.1	4.1±0.2	3.1±0.2	4.7±0.1	4.2±0.1	4.4±0.2	4.3±0.3	4.7±0.3	4.2±0.1	4.2±0.1
1	5.6±0.2	6.2±0.2	4.8±0.2	5.6±0.2	4.6±0.1	5.9±0.1	5.5±0.3	5.8±0.2	6.3±0.3	5.9±0.1	4.7±0.1	5.5±0.2
2	6.0±0.1	6.3±0.2	4.5±0.3	5.6±0.3	4.4±0.3	6.4±0.3	6.1±0.3	5.8±0.2	6.2±0.3	6.2±0.1	4.8±0.3	5.8±0.1
4	5.7±0.1	6.6±0.3	5.5±0.3	5.5±0.3	4.7±0.2	5.7±0.2	6.2±0.1	6.1±0.1	6.5±0.2	6.4±0.2	4.4±0.3	5.8±0.2
7	4.5±0.1	5.8±0.2	5.0±0.1	5.3±0.2	5.5±0.2	5.8±0.3	5.2±0.1	5.9±0.3	6.1±0.2	6.1±0.1	3.5±0.3	4.6±0.1
10	3.7±0.2	5.1±0.2	5.8±0.2	5.8±0.3	5.3±0.2	5.5±0.3	5.0±0.1	5.6±0.3	5.7±0.2	6.2±0.3	3.2±0.2	5.1±0.2
14	3.3±0.1	5.7±0.1	6.4±0.2	5.5±0.2	3.2±0.2	5.0±0.1	4.2±0.1	5.6±0.3	5.5±0.3	5.8±0.2	3.0±0.2	3.9±0.2
21	2.3±0.2	5.2±0.1	4.6±0.2	4.4±0.3	3.3±0.2	3.5±0.3	3.1±0.2	5.0±0.1	5.2±0.1	5.2±0.2	2.8±0.2	3.6±0.1
28	1.2±0.2	3.9±0.3	4.0±0.2	3.7±0.3	1.6±0.1	1.6±0.3	3.3±0.2	4.3±0.2	3.8±0.1	3.2±0.3	1.1±0.2	3.2±0.1
56	0.2±0.2	2.6±0.3	3.3±0.2	3.0±0.2	0.2±0.2	2.1±0.3	1.8±0.2	1.8±0.1	2.2±0.2	2.7±0.2	0.0±0.3	2.4±0.2
84	-0.5±0.1	1.9±0.3	1.1±0.2	1.8±0.2	-0.2±0.3	0.2±0.3	-0.1±0.3	0.6±0.2	1.4±0.3	1.2±0.3	-0.5±0.1	0.7±0.2
112	<-0.6 (2/3)[b]	0.8±0.3	<-0.6 (2/3)	0.2±0.2	<-0.6 (2/3)	-0.5±0.1		<-0.6 (2/3)	-0.2±0.3	<-0.6 (2/3)	<-0.6 (2/3)	-0.4±0.1
168				-0.3±0.3								<-0.6 (2/3)
210				<-0.6 (2/3)								

[a] Values presented as log CFU or MPN/g ± standard error; values are averages of a sample from each of the three replications (n=3)
[b] When samples fell below the limit of detection (<-0.6 log MPN/g), samples were enriched for the presence of *Salmonella*, presented in parentheses number positive for enrichment in 25 g out of the three samples.

Supplemental Table S5. Salmonella concentration (log CFU or MPN/g) by time point for each *Salmonella* strain in poultry-little amended clay-loam soils[a]

Time (Day)	4,12:i-	Branderup	Enteritidis	Javiana	Meleagridis	Montevideo	Muenchen	Newport E	Newport F	Paratyphi	Poona	Saintpaul
0	3.8±0.2	3.6±0.1	4.0±0.1	3.9±0.2	3.4±0.2	3.7±0.1	3.5±0.2	3.9±0.1	3.8±0.1	3.8±0.1	3.8±0.2	3.8±0.1
0.17	2.7±0.2	2.7±0.1	3.6±0.1	2.7±0.1	2.6±0.3	2.6±0.1	2.6±0.2	3.2±0.1	2.8±0.2	2.6±0.2	2.2±0.2	3.0±0.1
1	3.5±0.2	3.1±0.3	5.2±0.2	3.6±0.1	3.3±0.3	3.6±0.2	4.9±0.1	5.1±0.3	4.4±0.3	4.5±0.3	3.3±0.1	4.3±0.1
2	3.8±0.1	4.7±0.6	5.3±0.1	4.8±0.2	3.7±0.2	5.7±0.2	4.2±0.3	4.8±0.3	5.2±0.2	3.9±0.3	4.2±0.2	4.2±0.2
4	4.0±0.2	5.3±0.4	5.0±0.1	5.0±0.2	2.6±0.2	5.2±0.3	4.1±0.3	4.9±0.1	5.2±0.3	3.3±0.3	2.6±0.3	4.3±0.2
7	4.5±0.3	5.3±0.2	5.0±0.1	3.8±0.2	1.5±0.1	5.5±0.2	4.2±0.1	5.0±0.1	4.9±0.2	3.7±0.2	2.7±0.2	4.5±0.2
10	4.1±0.3	5.2±0.3	4.0±0.1	3.4±0.2	1.3±0.1	5.1±0.1	3.8±0.2	4.6±0.3	4.5±0.2	3.4±0.1	2.5±0.3	3.9±0.2
14	3.5±0.3	4.4±0.3	3.5±0.2	3.3±0.2	1.4±0.2	4.9±0.1	3.1±0.1	4.1±0.2	4.1±0.1	3.1±0.1	1.6±0.3	3.7±0.2
21	2.5±0.2	3.8±0.1	2.7±0.2	2.3±0.3	1.0±0.1	4.5±0.2	2.1±0.1	3.6±0.1	3.8±0.2	2.5±0.3	1.4±0.2	3.7±0.2
28	1.8±0.2	3.4±0.2	2.4±0.2	1.6±0.2	0.2±0.2	3.8±0.2	1.4±0.2	2.8±0.1	3.5±0.2	3.3±0.2	1.0±0.1	3.2±0.3
56	1.0±0.3	2.7±0.1	1.6±0.2	1.2±0.3	-0.5±0.1	2.3±0.2	1.0±0.1	2.3±0.2	2.3±0.2	2.2±0.1	0.6±0.1	2.4±0.2
84	<-0.6 E (3/3)[b]	2.3±0.1	0.8±0.1	0.4±0.3	<-0.6 E (3/3)	2.3±0.2	-0.2±0.3	1.4±0.2	1.3±0.2	1.5±0.2	<-0.6 H (3/3)	1.0±0.1
112	<-0.6 E (3/3)	1.6±0.3	<-0.6 F (3/3)	<-0.6 I (2/3)		0.9±0.2		-0.5±0.1	0.0±0.3	<-0.6 G (3/3)	<-0.6 H (3/3)	-0.5±0.1
168	<-0.6 E (2/3)[c]	0.2±0.2	<-0.6 F (2/3)			0.1±0.2		<-0.6 I (2/3)	<-0.6 G (3/3)	<-0.6 G (2/3)	<-0.6 H (2/3)	<-0.6 E (2/3)
210		<-0.6 I (2/3)				<-0.6 G (2/3)						

[a] Values presented as log CFU or MPN/g ± standard error; values are averages of a sample from each of the three replications (n=3)

[b] When samples fell below the limit of detection (<-0.6 log MPN/g), samples were enriched for the presence of *Salmonella*, presented in parentheses number positive for enrichment in 25 g out of the three samples
